# Co-occurrence probabilities between mosquito vectors of West Nile and Eastern equine encephalitis viruses using Markov Random Fields (MRFcov)

**DOI:** 10.1186/s13071-022-05530-1

**Published:** 2023-01-10

**Authors:** Mohamed F. Sallam, Shelley Whitehead, Narayani Barve, Amely Bauer, Robert Guralnick, Julie Allen, Yasmin Tavares, Seth Gibson, Kenneth J. Linthicum, Bryan V. Giordano, Lindsay P. Campbell

**Affiliations:** 1 Preventive Medicine and Biostatistics Department, Uniformed Service University of the Health Sciences, Bethesda, MD 20814 USA; 2 Department of Biology, University of Nevada, Reno, NV USA; 3 Whitehead Entomology Consulting, Gainesville, FL USA; 4 Manatee County Mosquito Control District, Palmetto, FL USA; 5 Department of Natural Resources, University of Florida, Gainesville, FL USA; 6 Florida Medical Entomology Laboratory (FMEL), Department of Entomology and Nematology, University of Florida Institute of Food and Agricultural Sciences (UF/IFAS), Gainesville, FL USA; 7 U.S. Department of Agriculture, Gainesville, FL USA

**Keywords:** Interspecies interaction, Spatial distribution, Conditional Markov Random Fields, Host-seeking mosquito, Community ecology

## Abstract

Mosquito vectors of eastern equine encephalitis virus (EEEV) and West Nile virus (WNV) in the USA reside within broad multi-species assemblages that vary in spatial and temporal composition, relative abundances and vector competence. These variations impact the risk of pathogen transmission and the operational management of these species by local public health vector control districts. However, most models of mosquito vector dynamics focus on single species and do not account for co-occurrence probabilities between mosquito species pairs across environmental gradients. In this investigation, we use for the first time conditional Markov Random Fields (CRF) to evaluate spatial co-occurrence patterns between host-seeking mosquito vectors of EEEV and WNV around sampling sites in Manatee County, Florida. Specifically, we aimed to: (i) quantify correlations between mosquito vector species and other mosquito species; (ii) quantify correlations between mosquito vectors and landscape and climate variables; and (iii) investigate whether the strength of correlations between species pairs are conditional on landscape or climate variables. We hypothesized that either mosquito species pairs co-occur in patterns driven by the landscape and/or climate variables, or these vector species pairs are unconditionally dependent on each other regardless of the environmental variables. Our results indicated that landscape and bioclimatic covariates did not substantially improve the overall model performance and that the log abundances of the majority of WNV and EEEV vector species were positively dependent on other vector and non-vector mosquito species, unconditionally. Only five individual mosquito vectors were weakly dependent on environmental variables with one exception, *Culiseta melanura*, the primary vector for EEEV, which showed a strong correlation with woody wetland, precipitation seasonality and average temperature of driest quarter. Our analyses showed that majority of the studied mosquito species’ abundance and distribution are insignificantly better predicted by the biotic correlations than by environmental variables. Additionally, these mosquito vector species may be habitat generalists, as indicated by the unconditional correlation matrices between species pairs, which could have confounded our analysis, but also indicated that the approach could be operationalized to leverage species co-occurrences as indicators of vector abundances in unsampled areas, or under scenarios where environmental variables are not informative.

**Graphical Abstract:**

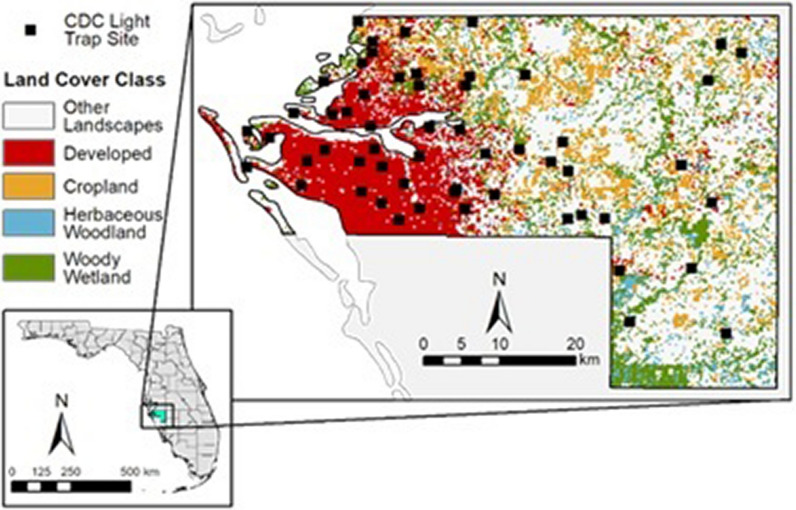

**Supplementary Information:**

The online version contains supplementary material available at 10.1186/s13071-022-05530-1.

## Background

Mosquito vector-borne pathogens such as eastern equine encephalitis virus (EEEV) and West Nile virus (WNV) are maintained and proliferate in the natural environment via a complex set of requirements and interactions with their hosts, underlying environmental variables and interactions with other organisms [1]. Both of these disease pathogens are autochthonously transmitted in the USA since their introduction in the 1990s and pose a continuous and consistent threat, particularly where multiple mosquito vectors are found. Although the inherent complexity of EEEV and WNV transmission systems is recognized broadly within community ecology [2], theoretical frameworks specific to disease ecology focus primarily on the biodiversity of vertebrate hosts and place much less emphasis on the biodiversity of insect vector species when considering arbovirus transmission dynamics [3–5]. However, mosquito vectors of these two pathogens reside within broader multi-species assemblages that vary in composition, abundances and vector competency to transmit EEEV and WNV, which can collectively impact pathogen transmission in a geographic area [6–10]. In Florida, the EEEV and WNV risk is a composite distributed across multiple species of the competent bridge and main mosquito vectors that may be sympatric but vary in abundance, diversity and vector competence [11–15]. More than 60 mosquito control districts (MCDs) conduct routine surveillance for these vector species to guide specific vector control efforts in Florida [16]. Unfortunately, vector control capabilities are limited and need to be carefully targeted toward the spatial and temporal distribution of mosquito vectors to be effective. Importantly, vector control techniques vary depending on the target mosquito species, which adds to the complexity of designing effective control programs against mosquito vectors of EEEV and WNV.

While range-wide distributions of single species are now routinely estimated using ecological niche models [17, 18], the distribution and abundance of species at local scales are likely to involve both meso- and microscale landscape features and the potential for interactions with other species that form the mosquito community. This complexity requires a different set of underlying data and analytical toolkits that can estimate both landscape factors and factors that promote or impede community co-occurrence. Due to challenges from both the data and analytical sides, work examining these factors in a single framework has remained piecemeal at best, with studies often focusing on just a subset of species and their possible interactions [17, 19, 20] or on larval distributions [21], not those of adults. However, virus transmission occurs in the adult life-stage of mosquitoes, thus investigating co-occurrence patterns of adult mosquitoes is essential for understanding transmission risk across geographic areas. Further, adult mosquito trapping data are collected routinely by MCDs, providing a means to scale-up analysis broadly.

A key challenge in predicting local-scale species distributions and community composition is accounting for covariance between species and the environment. Generalized additive models (GAMS) have been used to explore relationships between abundances of potential competitors in mosquito assemblages and a vegetation gradient [22], while other methods have focused on pairwise probability calculations between individual species [23–25], with some similarities across methods [26]. When considering landscape-scale co-occurrence, a particularly powerful and yet unused approach for mosquitoes is to first quantify correlations between species pairs and then determine whether the strength of these correlations is conditional on environmental variables using a conditional Markov Random Fields (CRF) analysis [25]. This approach simultaneously considers both biotic and abiotic factors that may be controlling the shape of species abundances, distributions and community composition across environmental gradients in space.

In this study, we leverage the capability of longitudinal collection data for mosquito communities, with the main emphasis on vectors of EEEV and WNV from Manatee County, Florida over the 2020 sampling season (May–December) to: (i) quantify correlations between host-seeking mosquito vector species of WNV and EEEV and other mosquito species, vectors and non-vectors; (ii) quantify correlations between host-seeking mosquito vectors and landscape and climate variables within their flying ranges; and (iii) investigate whether the strength of correlations between species pairs are conditional on landscape or climate variables using CRF analyses.

We hypothesized that species composition and abundances of WNV- and EEEV-competent mosquito species are most likely determined by co-occurrences between species pairs in specific landscape and/or climate features; that is, landscape features generally important for modifying species co-occurrence. Alternately, it may be that most vector mosquitoes are habitat generalists and generally co-occur regardless of landscape. The end goal of using this approach is to better understand the joint effects of landscape and other mosquito species drivers on mosquito diversity/density and provide data-driven information for more comprehensive management and control strategies.

## Methods

### Study area and mosquito collections

**Fig. 1:**
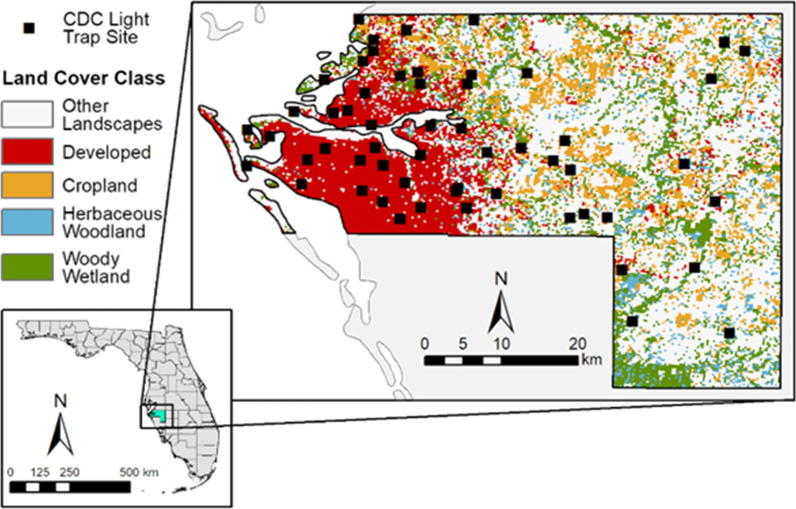
Study area with mosquito trap surveillance sites (filled squares) and investigated land cover classes in Manatee County, Florida, USA. CDC, US Centers for Disease Control and Prevention

Georeferenced 2020 mosquito trap data collected by Manatee County Mosquito Control District (MCMCD), Florida, were acquired from the VectorBase Bioinformatics Resource for Invertebrate Vectors of Human Pathogens repository (https://vectorbase.org; 2021). The MCMCD 2020 data resulted from collections using US Centers for Disease Control and Prevention (CDC) CO_2_-baited light traps set at 56 locations at weekly intervals from approximately May to December (Fig. 1). Although some trap and attractant biases exist, CDC CO_2_-baited light traps collect diverse mosquito species in Florida [27]. This was demonstrated by the mosquito species that were consistently collected in the 2020 MCMCD data set representing flood water, salt marsh and container-inhabiting mosquito communities. Light traps were set for approximately 12 h before sunset until dawn, and mosquito collections were identified to species by trained mosquito control personnel using the Darsie and Ward (2005) taxonomic key [28]. Species counts for each sampling week were recorded and formatted in Microsoft Excel (Microsoft Corp., Redmond, WA, USA) spreadsheets prior to submission to the VectorBase platform [29]. The mean number of mosquitoes per trap night per species was calculated at each trap site across 28 weeks during the 2020 sampling season, and a ‘site-by-species’ matrix was created with individual trap locations occupying rows and individual species occupying columns in preparation for analyses (Additional file 1: Data file S1).

### Vector-competent mosquito species

Laboratory-confirmed vector competency of mosquito vector species for EEEV and WNV were identified from the scientific literature (Table 1), based on the collected mosquito species from the MCMCD. The field-confirmed mosquito vectors of EEEV and WNV were also identified from previous studies and denoted as putative vector species in our study. We only included 17 WNV- and EEEV-competent vector species in our results and discussion. Other non-vector mosquito species are included in Additional file 2: Table S1.

**Table 1:** Unconditional correlations between laboratory-confirmed and two putative West Nile virus and eastern equine encephalitis virus vectors and other mosquito species within 5-km buffer distances

Vector species name^a^	Co-occurring species	Relative importance	5% quantile	Mean coefficient	95% quantile
*Aedes* *aegypti*^b^ [30]	*Mansonia titillans*^b,p^ [31]	0.320	− 0.044	− 0.044	− 0.044
*Aedes* *albopictus* [30, 32]	*Uranotaenia lowii*^b,p^ [31]	0.512	0.145	0.145	0.145
	*Psorophora* *ferox*	0.488	0.141	0.141	0.141
*Aedes* *atlanticus*^c^ [33, 34]	*Aedes* *infirmatus*^c^	0.944	0.459	0.459	0.459
*Aedes* *infirmatus*^c^ [35–39]	*Uranotaenia* *lowii*^b,p^	0.019	0.076	0.076	0.076
	*Psorophora* *ferox*	0.096	0.169	0.169	0.169
	*Anopheles* *crucians*^b^	0.151	0.212	0.212	0.212
	*Aedes* *fulvus pallens*^b^	0.996	− 0.059	− 0.059	− 0.059
*Aedes taeniorhynchus*^b^ [30]	*Culex* *iolambdis*	0.057	0.106	0.108	0.110
	*Aedes* *atlanticus*^b^	0.019	− 0.066	–0.062	− 0.059
*Aedes* *vexans *sensu lato^d^ [30, 40]	*Aedes* *infirmatus*^c^	0.070	0.042	0.047	0.120
	*Culex* *nigripalpus*	0.545	0.131	0.132	0.157
*Anopheles* *crucians*^b^ [41, 42]	*Culex* *erraticus*^c^	0.613	0.391	0.391	0.391
	*Aedes* *infirmatus*^c^	0.182	0.212	0.212	0.212
	*Culex* *nigripalpus*^b^ [43]	0.077	0.138	0.138	0.138
	*Anopheles* *quadrimaculatus*^c^	0.549	0.127	0.127	0.127
	*Manosonia* *titillans*	0.041	0.100	0.100	0.100
*Anopheles* *quadrimaculatus*^c^ [43]	*Culex* *erraticus* [44]	0.166	0.070	0.070	0.070
	*Coquillettidia* *perturbans*^d^ [38, 43]	0.124	0.060	0.060	0.0060
	*Culex*. *nigripalpus*^b^	0.113	0.058	0.058	0.058
	*Ur*ranotaenia *lowii*^b,p^	0.043	0.035	0.035	0.035
*Coquillettidia* *perturbans*^d^ [38, 43]	*Manosonia* *dyarii*	0.470	0.170	0.170	0.170
	*Culex* *erraticus*^c^	0.163	0.100	0.100	0.100
	*Anopheles* *quadrimaculatus*^c^	0.060	0.060	0.060	0.060
*Culex* *coronator*^b^ [45]	*Psorophora* *ferox*	0.593	0.177	0.177	0.177
*Culex*. *erraticus*^c^ [44]	*Masonia* *titillans*^b,p^	0.116	0.171	0.171	0.171
*Culex* *nigripalpus*^b^ [43]	*Psorophora* *columbiae*	0.175	0.146	0.146	0.147
	*Anopheles* *crucians*^b^	0.155	0.138	0.138	0.138
	*Culex* *erraticus*^c^	0.443	0.232	0.232	0.232
	*Aedes* *vexans s.*^d^	0.142	0.131	0.132	0.157
*Culex* *quinquefasciatus*^b^ [43]	*Culex* *coronator*^b^	0.381	0.129	0.129	0.129
*Culex* *restuans*^b^ [46]	*Culex* *quinquefasciatus*^b^ [43]	1	0.164	0.164	0.164

*P* Putative vector

^a^References for vector competency studies

^b^West Nile virus (WNV) vectors

^c^Eastern equine encephalitis virus (EEEV) vectors

^d^WNV and EEEV vectors

### Environmental data

US Geological Survey (USGS) Conterminous United States Land Cover Projections 1992–2100 were extracted for 2020 [47] and served as land cover data in our analyses. These data have a 250-m spatial resolution and consist of annual land cover classifications. We focused on four major land cover classifications found in Manatee County as representative of different levels of anthropogenic disturbance across the study area: developed, cropland, woody wetland and herbaceous wetland [48]. We quantified and extracted area percentages of each land cover type within both 5-km and 10-km buffers surrounding each mosquito trap location using the ‘landscape metrics’ package in R [49, 50].

Bioclimatic variables within buffer sets surrounding each trap site from 2020 daily ‘Parameter-elevation Regressions on Independent Slopes Model’ (PRISM) Climate Group data [51] were extracted at an 800-m spatial resolution using the ‘dismo’ package in R [52]. PRISM data were accessed from https://prism.oregonstate.edu/. To reduce the number of variables in our model, five bioclimatic variables were selected for analyses: Bio2 (mean diurnal temperature range), Bio5 (the maximum temperature of the warmest month), Bio9 (mean temperature of the driest quarter), Bio15 (precipitation seasonality) and Bio17 (precipitation of the driest quarter), based on mosquito biology and ecological data reported in previous studies [18, 21, 53]. Bioclimatic variables were all scaled to range between 0 and 1 in preparation for modeling given the widely different units in the raw data.

### Statistical analyses

We used CRFs executed in the ‘MRFcov’ (Markov Random Fields with additional covariates)package in R [25] to quantify whether the abundances of each WNV and EEEV mosquito vector species were (i) unconditionally dependent on another mosquito species in the assemblage and (ii) unconditionally dependent on a landscape or bioclimatic variable; and whether (iii) the strength level of dependence between species pairs was conditional on a landscape and/or bioclimatic variable or (iv) there were no correlations between species pairs nor between individual species and environmental variables. [25]. Accordingly, the unconditional correlations were estimated using the generated regression correlation matrices generated by the MRF and CRF analyses which refers to consistent correlation between either species pairs, or between individual vector species and environmental variables, with and without covariates using MRF and CRF analyses. Unlikely conditional correlations are: (i) correlation between species pairs only at specific habitats or climate which did not show unconditional correlations; (ii) correlations between single species and landscape and/or climate variables; or (iii) increased correlation strength between species pairs at specific habitats or climate in addition to their unconditional correlations, such as the correlation between *Coquillettidia perturbans* and *Mansonia dyarii* indicated by the generated regression correlation matrices (Tables 1, 3).

To prepare data for analysis, we rounded the mean mosquito per trap night value within our ‘site-by-species’ matrix to an integer value to serve as nodes (mosquito species) and added additional columns with percentage landscape and average bioclimatic variables to serve as the conditional variables in the model. To calculate CRF using abundance data, the ‘MRFcov’ package log-transforms species counts before performing pairwise linear regressions across all combinations of species and environmental variables, using an optimized regularization multiplier for variable selection and to reduce overfitting; predicted and observed values for all species combinations are then used in model evaluation [25]. Geographic coordinates at each mosquito trap location were included to fit a spatial spline to account for residual spatial autocorrelation that can inflate parameter estimates resulting in Type I errors [54]. Bootstrap spatial models analyses using 500 replicates and 100% of sampling points with random replacement in each replicate were used to capture uncertainty in parameter estimates [25], and key regression coefficients of each species were output to a single table showing the relative importance of each variable with a threshold value of > 0.01 and mean coefficient values (Additional file 1: Data file S1). The relative importance values indicate the relative strength of a variable on the log abundance of a vector species out of all variable combinations calculated for the species, while the sign of the mean coefficient values shows the direction of these correlations. Two separate models were run within each of the 5-km and 10-km buffer distances from each trap location: one model with and one model without environmental variables.

## Results

Manatee County, Florida, is located on the western coast of the Florida Peninsula on the Gulf of Mexico (Fig. 1). Along the coast, the area is primarily covered by developed land, while the inland extent of the county is predominantly rural and consists of agricultural land interspersed with wetlands [47]. The region is characterized by a humid subtropical climate [55] with average annual maximum temperatures in the range of 27 °C to 29 °C and average annual minimum temperatures in the range of 15 °C to 17 °C (Florida Climate Prediction Center [CPC] prediction maps CCPM 2022) [056]. The average annual precipitation ranges from around 1250 to 1400 mm [57], with most of it falling during the rainy season, which typically lasts from May to October.

**Fig. 2:**
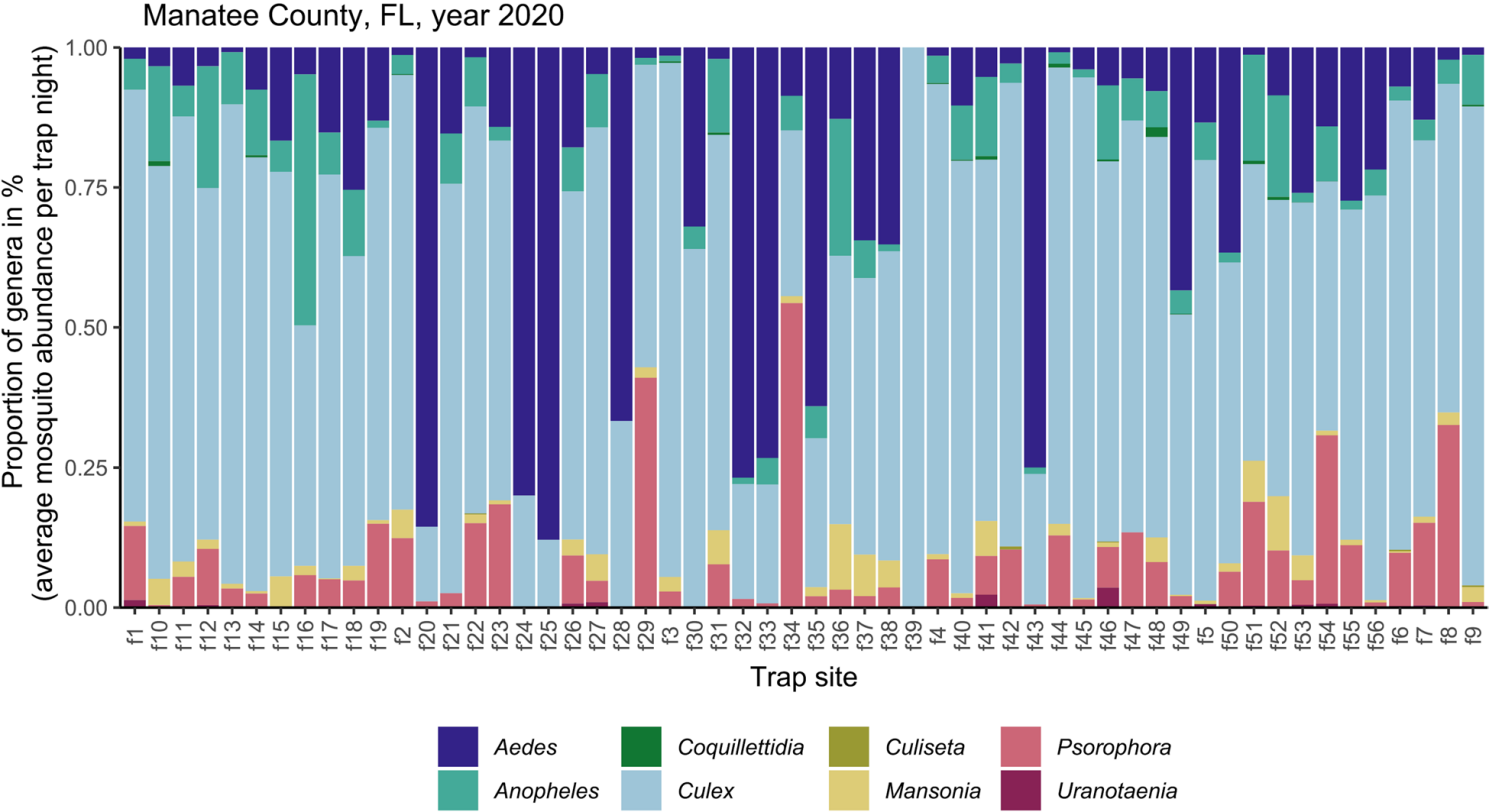
Proportions of mosquito genera across 2020 trap collections in Manatee County, FL

A total of 2,009,985 adult female mosquitoes representing 30 species and eight genera were collected across 56 trap sites during the 2020 mosquito trap surveillance sampling from May to December. Initial exploration of mosquito abundances by genera across the trap sites indicated variability in the abundance of mosquito genera across these sites. *Culex* was the dominant genus found across these sites; however, approximately 14% of sites were dominated by *Aedes* mosquitoes (Fig. 2).

### Statistical tests

**Fig. 3:**
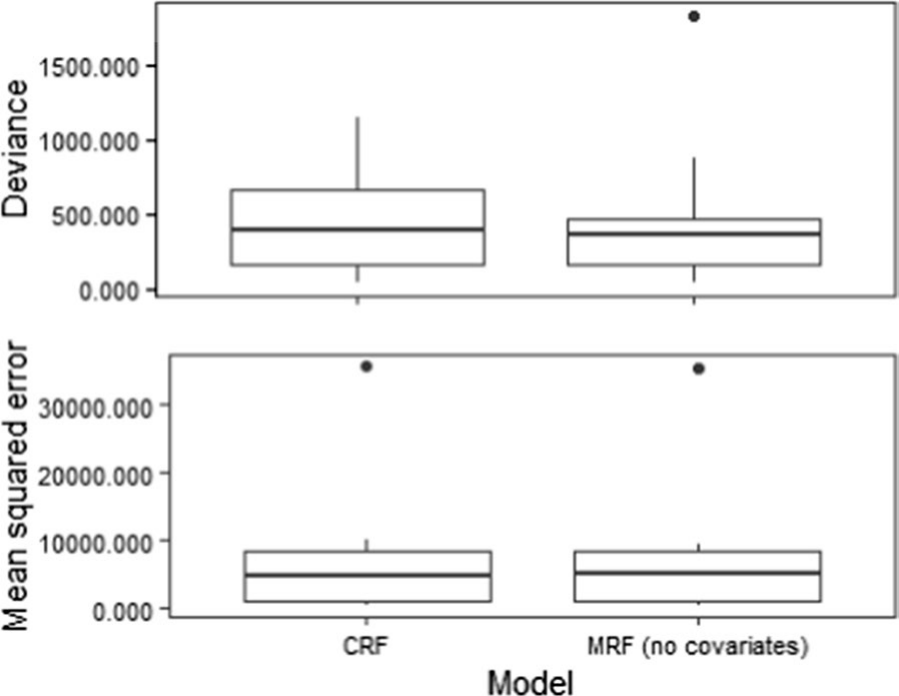
Box plots of CRF analyses with covariates (left) and MRF analysis without covariates (right) show mean squared error (MSE) and deviance within 5 km. CRF, Conditional Markov Random Fields; MRF, Markov Random Fields

**Fig. 4:**
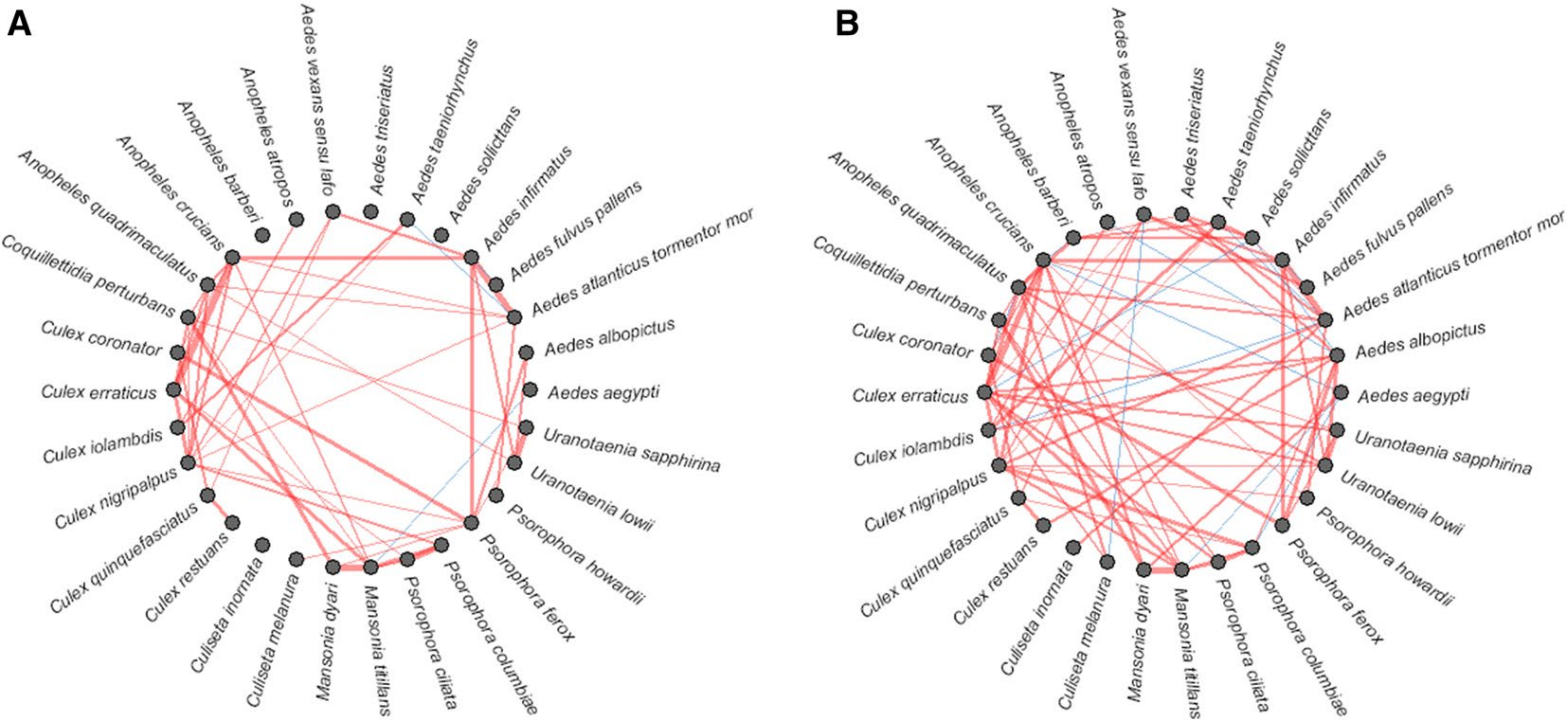
Network correlations between highly connected species pairs without (**A**) and with (**B**) environmental covariates measured within a 5-km buffer show the change in the number and direction of correlations between species pairs by adding all environmental covariates

Box plots for bootstrapped models with no covariates (MRF) and with covariates (CRF) measured within 5-km and 10-km buffer distances indicated that the inclusion of landscape and bioclimatic variables did not substantially improve the overall model performance when evaluated by deviance (DV) or mean squared error (MSE) values (Fig. 3; Additional file 1: Figure S1). The insignificant differences in DV and MSE values reflect that the change in number and strength of correlations between species pairs, as indicated by relative importance values, did not change the overall spatial correlations between mosquito species pairs. This insignificant small change in correlations between species pairs without (Fig. 4) and with environmental variables (Fig. 4), as demonstrated in network plots, showed that the number and direction of correlations between individual species pairs were slightly impacted by the environmental variables included in the model. The plot shown in Fig. 4b demonstrates correlations between species pairs that were not affected by the inclusion of environmental variables and correlations between species pairs where the strength of correlation varies with the inclusion of an environmental variable. The reduced number of species pairs in the plot shown in Fig. 4b compared to that shown in Fig. 4a indicated that several species pairs were highly correlated when no environmental variables were included in the model but that these correlations reduced to zero when environmental variables were added.

Model regression correlation matrices included key coefficient tables that summarized the relative importance of correlations between log abundances of species pairs or between the log abundance of an individual vector species and environmental variables (i.e. unconditional correlations with another species measured within 5-km and 10-km buffer distances), or conditional correlations between species pairs where the strength of the correlation changes in specific land cover classes or climate conditions measured within 5-km and 10 km-buffer distances (Tables 1, 3: 5-km buffer; Additional file 2: Table S1; Additional file 3: Table S2 for all species within 5 km and 10 km). The corresponding mean coefficient values derived across the 500 bootstrapped model replicates, using random replacement in each replicate, with 5% and 95% quantiles provide a measure of uncertainty. Key coefficient values for all species combinations with relative importance values > 0.01 are available in Additional file 2: Table S1; Additional file 3: Table S2.

Overall, the regression coefficients shown in Tables 1–3 indicated that a greater number of vector species were unconditionally correlated with another mosquito vector species than with the environment; however, some species did exhibit correlations with landscape and climate variables, and several species pairs were no longer correlated with one another when environmental variables were added. The log abundances for 16 of the 20 WNV and EEEV vector species investigated were unconditionally correlated with another mosquito vector or non-vector species (Table 1). Also, five vector species demonstrated conditional dependence on environmental variables (Table 2). Out of these five vectors, three WNV vector species, one EEEV vector species and one vector species of both WNV and EEEV were conditionally dependent on three climates (Bio2, Bio9, Bio15) and two landscape variables (developed and woody wetland) measured within a 5-km buffer distance (Table 2). For example, *Culiseta melanura*, the primary vector for EEEV, only showed a strong conditional correlation with precipitation seasonality (Bio15; relative importance = 0.446), average temperature of driest quarter (Bio 9; relative importance = 0.303) and woody wetland (relative importance = 0.226), and not with other mosquito species (Table 2). Results from models including environmental variables measured within 10-km buffer distances indicated that five vector species showed conditional correlations with four climate variables (Bio2, Bio5, Bio9, Bio17) and one landscape (developed) variable (Additional file 3: Table S2).

**Table 2:** Conditional correlations between vector species and environmental variables within 5-km buffer radii

Vector species name	Environmental variable	Relative importance	5% quantile	Mean coefficient	95% quantile
*Aedes* *aegypti*^a^	Bio9 (Mean temperature of driest quarter)	0.680	0.064	0.064	0.064
*Aedes* *taeniorhynchus*^a^	Bio2 (Mean diurnal range)	0.903	− 0.430	− 0.430	− 0.429
*Aedes* *vexans* *s*.*l*.^b^	Bio9 (Mean temperature of driest quarter)	0.382	− 0.166	− 0.110	− 0.110
*Culex* *coronator*	Developed	0.027	0.038	0.038	0.038
*Culiseta melanura*^c^ [55, 58]	Bio9 (Mean temperature of driest quarter)	0.303	− 0.035	− 0.035	-0.035
	Bio15 (Precipitation seasonality)	0.446	0.042	0.042	0.042
	Woody wetland	0.226	0.030	0.030	0.030

^a^WNV vectors

^b^WNV and EEEV vectors

^c^EEEV vectors

We found limited evidence for conditional correlations between species pairs where the strength of correlations between these pairs changes in a specific landscape or climate variable, in both 5-km (Table 3) and 10-km buffer distances (Additional file 3: Table S2). Models run within a 5-km buffer distance indicated this category of conditional correlations between only three species pairs in specific habitats (cropland, woody wetland, herbaceous wetland) (Table 3) where at least one species in the species pairs is a vector. The relative importance of environmental variables on conditional correlations increased within the 10-km buffer distance, with nine species pairs demonstrating conditional correlations varying with landscape and climate variables. While climate variables did not have any effect on the strength of conditional correlation between species pairs within 5 km (Table 3), climate variables impacted the strength of conditional correlations between seven species pairs within 10-km buffer radii (Additional file 3: Table S2).

**Table 3:** Conditional correlations between species pairs where the strength of correlation varied with environmental variables within 5-km buffer radii

Name of species 1	Name of species 2	Variable	Relative importance	5% quantile	Mean coefficient	95% quantile
*Coquillettidia* *perturbans*^a^	*M*ansonia *dyarii*	Cropland	0.286	0.132	0.132	0.132
*Culex* *coronator*^a^	*Anopheles* *crucians*^a^	Woody wetland	0.053	− 0.053	− 0.053	− 0.053
*Culex* *nigripalpus*^a^	*Aedes* *taenorhynchus*^a^	Herbaceous wetland	0.026	− 0.056	− 0.056	− 0.056

^a^WNV vectors

## Discussion

The diversity of host-seeking mosquito vectors with different feeding preferences and their spatial and temporal co-occurrences have been highlighted in previous studies to play an important role in the circulation, maintenance and transmission of disease pathogens in mosquito populations [6, 8, 10]. In this study, we investigated abundances of known and putative WNV and EEEV vector species using a community ecology approach that quantified correlations with other vector and non-vector mosquito species, as well as with landscape and climate variables, and then determined if and how the strength of correlations between species pairs change across environments. The result is a novel view of mosquito vector occurrence in the context of abiotic and community factors and highlights the potential to use species co-occurrences as indicators of vector abundances in the absence of direct observations, or under scenarios where environmental variables are not informative.

Based on previous empirical observations linking mosquito vector abundances with environmental variables [59, 60], we expected to find that log abundances of vector species would be strongly correlated with the landscape and climate variables. Surprisingly, our results indicated that the log abundances of 10 out of 13 WNV vector species, three out of four EEEV vector species and three vector species for both WNV and EEEV were positively correlated with other mosquito species, but only weakly correlated or not correlated at all with environmental variables. We only found three cases of negative correlation between species: *Aedes aegypti* and *Mansonia titillans* (mean coefficient value = − 0.044), *Aedes infirmatus* and *Aedes fulvus pallens* (mean coefficient value = − 0.059), and *Aedes taeniorhynchus* and *Aedes atlanticus* (mean coefficient value = − 0.059), indicating low log abundances of the former species of each pair at collection sites were associated with high abundances of the respective latter species (Table 1).

A challenge with interpreting co-occurrence results is how to link those to the underlying mechanisms. Co-occurrence can provide a basis for more detailed studies attempting to demonstrate direct biotic interactions. It may also be that co-occurrence instead reflects differential micro-scale habitats not fully captured in the abiotic variables used. In the example above of a negative co-occurrence of *Ae. aegypti* and *Ma. titillans*, *Ae. aegypti* prefers water containers in urban areas [20, 61, 62], whereas *Ma. titillans* requires more permanent freshwater with emergent aquatic vegetation [53, 63]. Given that our models were estimated for host-seeking mosquitoes, within their flight ranges, and do not fully capture the microscale habitat preferences, the negative co-occurrence may simply be due to this microscale patterning, rather than, for example, direct competition.

The correlation of WNV and EEEV vectors with other species and less with environmental variables, as shown in our results, may indicate: (i) geographic overlapping, due to small study area, in the flight ranges between the studied mosquito species, and/or (ii) that some of these vectors are typically broad-habitat generalists, which can present challenges when investigating occurrence patterns using environmental variables alone. For example, the strong co-occurrence between *Culex restuans* and *Culex quinquefasciatus* and moderate co-occurrence between *Aedes vexans* and *Ae. infirmatus* may again reflect broad occurrence across landscape types. In addition, the potential for unmeasured covariance between spatial and temporal niche dynamics, especially given these taxa are known to be tied to the dynamics of wet season timing in North and Central Florida, may contribute to observed patterns [53, 64].

Our modeling approach does clearly delineate some broad-scale habitat specialists. For example, *Cs. melanura*, the primary enzootic vector of EEEV, was strongly correlated with landscape and bioclimatic variables, but not with other mosquito species. Compared to the more generalist vectors in our study area, *Cs. melanura* is a known specialist species with a strong preference for hardwood swamps as breeding habitats [65], and our results are consistent with those of previous studies that associated this species with woody wetland [66–68]. Another species, *Ae. taeniorhynchus*, was most abundant in areas with low mean diurnal temperature ranges, which almost certainly reflects its coastal affinities [69–71], where residual heating or cooling from ocean temperatures reduces onshore fluctuations in temperatures and increases water salinity.

Variation in the strength and direction of dependence between pairs of vector species across different environmental variables was of particular interest in terms of the goal of providing more comprehensive information about habitats in which multiple vector species may occur. Only one vector species, *Cq. perturbans*, demonstrated such conditional correlations. The shifts in the strength of mean coefficient values between *Cq. perturbans* and *Ma. dyarii* was affected by cropland habitats. The positive correlation between *Cq. perturbans* and *Ma. dyari* in croplands specifically, including wooded areas, reflects the importance of these habitats in predicting the level and strength of correlations between the two species compared to their unconditional correlations with each other in other habitats [72–75]. This conditional correlation brings home that climatic and land-use changes may differentially shift risks for different disease vector abundances such as, for example, shifts in dry quarter precipitation differentially favoring *Culex. nigripalpus* (a competent WNV vector) at the expense of *Ae. taeniorhynchus* (a less competent WNV vector).

We observed insignificant differences between model performance with and without environmental variables for co-occurrences between species pairs. We also observed only slight variation in model results when comparing effects of environmental variables measured within 5-km buffer distances and within 10-km buffer distances, as indicated by the relative importance values. However, increasing our buffer radius from 5 to 10 km resulted in a slight increase in the number of vector species demonstrating correlations with another mosquito species only, and not with environmental variables, from 32 to 34 pairs. Increased buffer extents capture greater mosquito communities and potential variability in climate and landscape conditions, which may be only marginally variable across smaller geographic areas such as Manatee County. Considerations of scale in the use of such approaches are particularly important to consider, especially given our discussion above regarding complexities with interpreting co-occurrence (or co-abundance) in relation to (here unmeasured) microhabitat drivers.

Although the collected mosquito vector diversity in the current study was robust, additional longitudinal data of mosquito collections, which could include other sampling techniques such as ovitraps, are needed to capture intra- and inter-annual population fluctuations between species pairs and to investigate additional environmental covariates at different resolutions across space and time. Moreover, the conditional correlations between host-seeking disease-vector species and other species not involved in the transmission of pathogens in specific habitats and climate conditions need further investigation to identify variation in both intra- and inter-seasonal correlations using a robust data collection across time and space. Additionally, because our study focused on host-seeking female mosquitoes, further investigation into the contribution of mosquito flight distances and their contributions to observed patterns is warranted. The purpose of this study was not to dissect the underlying processes and mechanisms that determine community abundances across our study area; however, our results highlight points of interest for continued investigation in the context of understanding underlying transmission risk. Specifically, continued investigation into the contributions of competition/exclusion in mature and immature habitats and the role of such biotic interactions in the distribution of vector mosquitoes will be critical.

## Conclusion

The landscape and bioclimatic covariates did not substantially improve the overall model performance, and the majority of WNV and EEEV vector species were positively correlated with other vector and non-vector mosquito species. This may reflect: (i) the small geographic size of the study area with less environmental heterogeneity and that distances between habitats are within the foraging range of most of mosquito species; (ii) that mosquito abundance and distribution in our study sites are predicted by the biotic factors (here unmeasured) in the water habitats, such as abundance of other mosquito species, and not climate; and/or (iii) that the mosquito community in Manatee County is habitat generalist, according to literature from similar studies. Only one exception, *Culiseta melanura*, the primary vector for EEEV, showed a strong conditional correlation with woody wetland, precipitation seasonality and average temperature of driest quarter, but not any other species. Some of the studied mosquito vector species are habitat generalists, indicated by a low number of conditional correlations with environmental variables but which also indicated that the approach could be operationalized to leverage species co-occurrences as indicators of vector abundances in unsampled areas, or under scenarios where environmental variables are not informative. Also, considerations of geographic scale in the use of CRF approach are particularly important to be addressed in future studies to explain the complexities of co-occurrence (or co-abundance) in relation to microhabitat drivers.

## Supplementary Information

**Additional file 1: Data file S1. ** Data and r code developed for the analysis (https://github.com/slmmhm/MRFcov-Manatee-county.git). **Figure S1.** Box plots of MRF analyses with covariates (left) and without covariates (right) show MSE and deviance within 10 km.

**Additional file 2: Table S1.** Key coefficient values derived from 500 bootstrap replicates for models run with environmental covariates measured within a 5-km distance from trap locations. SpName is the mosquito species response variable, Variable is the explanatory variable and rows with two variables separated by an "_" indicate conditional dependence. Rel_importance represents the relative importance of the Variable on the log abundance of the SpName count out of all combinations of variables for the individual SpName. Lower is the lower 10% confidence level, Mean_coef is the mean coefficient value and Upper is the upper 90% confidence value. Rows with NA values indicate that the log abundances SpName did not demonstrate dependence on another variable (species or environmental variable) included in the data set.

**Additional file 3: Table S2.** Key coefficient values derived from 500 bootstrap replicates for models run with environmental covariates measured within a 10-km distance from trap locations. SpName is the mosquito species response variable, Variable is the explanatory variable and rows with two variables separated by an "_" indicate conditional dependence. Rel_importance represents the relative importance of the Variable on the log abundance of the SpName count out of all combinations of variables for the individual SpName. Lower is the lower 10% confidence level, Mean_coef is the mean coefficient value and Upper is the upper 90% confidence value. Rows with NA values indicate that the log abundances SpName did not demonstrate dependence on another variable (species or environmental variable) included in the data set.

## Data Availability

The data presented, and the working code used in this study are available in Additional file 1: S1.

## Abbreviations

CDC: US Centers for Disease Control and Prevention
CRF: Conditional Markov Random Fields
EEEV: Eastern equine encephalitis virus
MCDs: Mosquito control districts
MCMCD: Manatee County Mosquito Control District
MRFcov: Markov Random Fields with additional covariates
WNV: Western Nile virus

## References

[1] Pavlovskiĭ EN (1966). Natural nidality of transmissible diseases, with special reference to the landscape epidemiology of zooanthroponoses.

[2] Collinge SK, Ray C (2006). Disease ecology: community structure and pathogen dynamics.

[3] Keesing F (2010). Impacts of biodiversity on the emergence and transmission of infectious diseases. Nature.

[4] Kilpatrick AM (2006). West Nile virus epidemics in North America are driven by shifts in mosquito feeding behavior. PLoS Biol.

[5] Schmidt S, Ostfeld R (2001). Biodiversity and the dilution effect in disease ecology. Ecology.

[6] Franklinos LHV (2019). The effect of global change on mosquito-borne disease. Lancet Infect Dis.

[7] Johnson PT, de Roode JC, Fenton A (2015). Why infectious disease research needs community ecology. Science.

[8] Lord C (2009). The effect of multiple species on arbovirus transmission. Israeli J Ecol Evol.

[9] McMillan JR, Armstrong PM, Andreadis TG (2020). Patterns of mosquito and arbovirus community composition and ecological indexes of arboviral risk in the northeast United States. PLoS Negl Trop Dis.

[10] Roche B (2013). The impact of community organization on vector-borne pathogens. Am Nat.

[11] Lee JH (2002). Identification of mosquito avian-derived blood meals by polymerase chain reaction-heteroduplex analysis. Am J Trop Med Hyg.

[12] Komar N (2003). Experimental infection of North American birds with the New York 1999 strain of West Nile virus. Emerg Infect Dis.

[13] Cupp EW (2004). Identification of reptilian and amphibian blood meals from mosquitoes in an Eastern equine encephalomyelitis virus focus in central alabama. Am J Trop Med Hyg.

[14] Molaei G (2007). Host feeding pattern of culex quinquefasciatus (diptera: culicidae) and its role in transmission of West Nile virus in harris county, texas. Am J Trop Med Hyg.

[15] Osorio HC, Ze-Ze L, Alves MJ (2012). Host-feeding patterns of culex pipiens and other potential mosquito vectors (diptera: culicidae) of West Nile virus (flaviviridae) collected in portugal. J Med Entomol.

[16] Lloyd A, Connelly C, Carlson D. Florida Mosquito Control: the state of the mission as defined by mosquito controllers, regulators, and environmental managers. Vero Beach, FL: Florida Coordinating Council on Mosquito Control; 2018

[17] Benedict MQ (2007). Spread of the tiger: global risk of invasion by the mosquito *Aedes albopictus*. Vect Borne Zoonotic Dis.

[18] Kraemer MU, et al. The global distribution of the arbovirus vectors *Aedes aegypti* and *Ae. albopictus*. eLife. 2015;4. 10.7554/eLife.08347 PMC4493616 26126267

[19] Bargielowski IE, Lounibos LP, Carrasquilla MC (2013). Evolution of resistance to satyrization through reproductive character displacement in populations of invasive dengue vectors. Proc Natl Acad Sci USA.

[20] Hopperstad KA, Reiskind MH (2016). Recent changes in the local distribution of aedes aegypti (diptera: culicidae) in South Florida, USA. J Med Entomol.

[21] Hopperstad KA, Sallam MF, Reiskind MH (2020). Estimations of fine-scale species distributions of *Aedes aegypti* and *Aedes albopictus* (diptera: culicidae) in Eastern Florida. J Med Entomol.

[22] Jansen F, Oksanen J (2013). How to model species responses along ecological gradients—Huisman–Olff–Fresco models revisited. J Veg Sci.

[23] Veech JA (2013). A probabilistic model for analysing species co-occurrence. Glob Ecol Biogeogr.

[24] Veech JA (2014). The pairwise approach to analysing species co-occurrence. Glob Ecol Biogeogr.

[25] Clark NJ, Wells K, Lindberg O (2018). Unravelling changing interspecific interactions across environmental gradients using Markov random fields. Ecology.

[26] Arita HT (2016). Species co-occurrence analysis: Pairwise versus matrix-level. Glob Ecol Biogeogr.

[27] Giordano BV (2020). Mosquito community composition, seasonal distributions, and trap bias in Northeastern Florida. J Med Entomol.

[28] Darsie RF, Ward RA (2005). Identification and Geographical distribution of the mosquitos of North America, North of Mexico.

[29] Rund S, et al. MIReAD, a minimum information standard for reporting arthropod abundance data. Scientific Data. 2019;6. 10.1038/s41597-019-0042-5 PMC6484025 31024009

[30] Turell MJ (2001). Vector competence of North American mosquitoes (Diptera: Culicidae) for West Nile virus. J Med Entomol.

[31] Unlu I (2010). Detection of West Nile virus RNA in mosquitoes and identification of mosquito blood meals collected at alligator farms in Louisiana. J Med Entomol..

[32] Turell MJ (1994). Experimental transmission of eastern equine encephalitis virus by strains of Aedes albopictus and A. taeniorhynchus (Diptera: Culicidae). J Med Entomol..

[33] Godsey MS, King RJ, Burkhalter K, Delorey M, Colton L, Charnetzky D (2013). Ecology of potential West Nile virus vectors in Southeastern Louisiana: enzootic transmission in the relative absence of Culex quinquefasciatus. Am J Trop Med Hyg..

[34] Baqar S, Hayes CG, Murphy JR, Watts DM (1993). Vertical transmission of West Nile virus by Culex and Aedes species mosquitoes. Am J Trop Med Hyg..

[35] Wellings FM, Lewis AL, Pierce LV (1972). Agents encountered during arboviral ecological studies: Tampa Bay area, Florida, 1963 to 1970. Am J Trop Med Hyg..

[36] Cupp EW (2003). Transmission of Eastern Equine Encephalomyelitis virus in central Alabama. Am J Trop Med Hyg..

[37] Hassan HK (2003). Avian host preference by vectors of Eastern Equine Encephalomyelitis virus. Am J Trop Med Hyg..

[38] Vaidyanathan R (1997). Vector competence of mosquitoes (Diptera:Culicidae) from Massachusetts for a sympatric isolate of Eastern Equine Encephalomyelitis virus. J Med Entomol..

[39] Florida Department of Health annual report. 2010. https://www.floridaheath.gov/licensing-and-regulation/reports-and-publications/_documents/09-10mqa-ar.pdf. Accessed Mar 2022.

[40] Turell MH (2005). An update on the potential of north American mosquitoes (Diptera: Culicidae) to transmit West Nile Virus. J Med Entomol..

[41] Gubler DJ (2007). Fields Virology.

[42] Mackay AJ. Detection of West Nile virus activity in male and female mosquitoes, and evaluation of host-utilization patterns of mosquitoes, in East Baton Rouge Parish, Louisiana. 2007. LSU Doctoral Dissertations. p. 3444.

[43] Sardelis MR (2001). Vector competence of selected North American Culex and Coquillettidia mosquitoes for West Nile virus. Emerg Infect Dis..

[44] Bingham AM (2016). Vector competence and capacity of Culex erraticus (Diptera: Culicidae) for Eastern Equine Encephalitis virus in the Southeastern United States. J Med Entomol..

[45] Alto BW (2014). Reproductive biology and susceptibility of Florida Culex coronator to infection with West Nile virus. Vector Borne Zoonotic Dis.

[46] Ebel GD (2005). Culex restuans (Diptera: Culicidae) relative abundance and vector competence for West Nile Virus. J Med Entomol..

[47] Sohl T, et al. Conterminous United States land cover projections-1992 to 2100. US Geological Survey data release. 2018. 10.5066/P95AK9HP.

[48] Volk M, et al., Florida land use and land cover change in the past 100 years. Florida’s climate: changes, variations, and impacts. 2017. http://purl.flvc.org/fsu/fd/FSU_libsubv1_scholarship_submission_1515440747_56b1ed92. Accessed July 2021.

[49] Hesselbarth M (2019). Landscapemetrics: an open-source R tool to calculate landscape metrics. Ecography.

[50] R Core Team, R: A language and environment for statistical computing. Vienna, Austria: R Foundation for Statistical Computing. 2021. ISBN 3-900051-07-0.

[51] Daly C (2001). High-quality spatial climate data sets for the United States and beyond. Transact Am Soc Agric Eng.

[52] Hijmans R, et al., Dismo: species distribution modeling. r package version 1.3–3. 2020. https://CRAN.R-project.org/package=dismo. Accessed Aug 2022.

[53] Sallam MF (2016). Ecological niche modeling of mosquito vectors of West Nile virus in St. John’s County, Florida, USA. Parasit Vectors.

[54] Banerjee S, Carlin BP, Gelfand AE (2004). Hierarchical modeling and analysis for spatial data.

[55] Kottek M (2006). World map of the Köppen-Geiger climate classification updated. Meteorol Z.

[56] https://www.cpc.ncep.noaa.gov/.

[57] Winsberg MD (2003). Florida weather.

[58] Andreadis TG, Anderson JF, Tirrell-Peck SJ (1998). Multiple isolations of eastern equine encephalitis and highlands J viruses from mosquitoes (Diptera: Culicidae) during a 1996 epizootic in southeastern Connecticut. J Med Entomol..

[59] Sallam MF (2017). Systematic review: land cover, meteorological, and socioeconomic determinants of Aedes mosquito habitat for risk mapping. Int J Environ Res Public Health.

[60] Mullen GR, LA Durden. Medical and veterinary entomology. 3rd ed. Cambridge: Academic Press; 2019. p. 3. ISBN: 9780128140437.

[61] Hribar LJ (2004). Mosquito larvae (Culicidae) and other diptera associated with containers, storm drains, and sewage treatment plants in the florida keys, Monroe County. Florida Entomologist.

[62] Reiskind MH, Lounibos LP (2009). Effects of intraspecific larval competition on adult longevity in the mosquitoes *Aedes aegypti* and *Aedes albopictus*. Med Vet Entomol.

[63] Linley JR, Linley PA, Lounibos LP (1986). Light and scanning electron microscopy of the egg of Mansonia titillans (diptera: culicidae)12. J Med Entomol.

[64] Ferreira-de-Freitas L (2020). An evaluation of characters for the separation of two culex species (diptera: culicidae) based on material from the upper midwest. J Insect Sci.

[65] Scott TW, Weaver SC. Eastern equine encephalomyelitis virus: epidemiology and evolution of mosquito transmission. In: Maramorosch K, Murphy FA, Shatkin AJ, editors. Advances in virus research. Cambridge: Academic Press; 1989. p. 277–328. 10.1016/s0065-3527(08)60838-6 2574935

[66] Morris CD (1980). Epizootiology of eastern equine encephalomyelitis virus in upstate New York, USA. I. introduction, demography and natural environment of an endemic focus. J Med Entomol.

[67] Morris CD, Zimmerman RH, Edman JD (1980). Epizootiology of eastern equine encephalomyelitis virus in upstate New York, USA. II. population dynamics and vector potential of adult Culiseta melanura (diptera: culicidae) in relation to distance from breeding site. J Med Entomol.

[68] Howard JJ, White DJ, Muller SL (1989). Mark-recapture studies on the culiseta (diptera: culicidae) vectors of eastern equine encephalitis virus. J Med Entomol.

[69] Fehring WK. Data bases for use in fish and wildlife mitigation planning in tampa bay, Florida: project summary. 1986. https://www.tampabay.wateratlas.usf.edu/upload/documents/DataBasesUseFishWildlifeMitigationPlanTampaBay.pdf. Accessed July 2021.

[70] Shaman J (2002). Using a dynamic hydrology model to predict mosquito abundances in flood and swamp water. Emerg Infect Dis.

[71] Qualls WA (2021). Shift in the spatial and temporal distribution of *Aedes taeniorhynchus* following environmental and local developments in St. Johns County Florida. Wetlands Ecol Manag.

[72] Baqar S (1993). Vertical transmission of West Nile virus by Culex and Aedes species mosquitoes. Am J Trop Med Hyg.

[73] Sardelis MR (2001). Vector competence of selected North American Culex and Coquillettidia mosquitoes for West Nile virus. Emerg Infect Dis.

[74] Farajollahi A (2009). Field efficacy of BG-sentinel and industry-standard traps for Aedes albopictus (diptera: culicidae) and West Nile virus surveillance. J Med Entomol.

[75] Pereira-Dos-Santos T (2020). A systematic review: is aedes albopictus an efficient bridge vector for zoonotic arboviruses. Pathogens.

