# Supplementary material for: Co-occurrence probabilities between mosquito vectors of West Nile and Eastern equine encephalitis viruses using Markov Random Fields (MRFcov)
Source: Parasit Vectors. 2023 Jan 10;16:10. doi: 10.1186/s13071-022-05530-1 (PMC9830877; doi:10.1186/s13071-022-05530-1)
Supplement: Supplementary file 1 — Additional file 1: Data file S1. Data and r code developed for the analysis (https://github.com/slmmhm/MRFcov-Manatee-county.git). Figure S1. Box plots of MRF analyses with covariates (left) and without covariates (right) show MSE and deviance within 10 km. [file 13071_2022_5530_MOESM1_ESM.docx]

Additional file

S1 Data and r code developed for the analysis “<https://github.com/slmmhm/MRFcov-Manatee-county.git>”


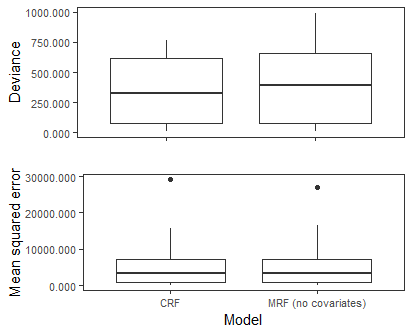


S2 Figure.  Box plots of MRF analyses with (left) and without covariates (right) show MSE and deviance within 10 Km.

S3a &b Tables: Provided separately in spreadsheet
